# Population structure of Plains Sucker *Pantosteus jordani* (Evermann 1893) in Canada

**DOI:** 10.1371/journal.pone.0355547

**Published:** 2026-08-26

**Authors:** Linda Lait, Doug A. Watkinson, Abdullah A. Gharamah, Patrick C. Hanington, Mark Poesch

**Affiliations:** 1 Department of Biological Sciences, Algoma University, Sault Ste. Marie, Ontario, Canada; 2 Freshwater Institute, Fisheries and Oceans Canada, Winnipeg, Manitoba, Canada; 3 Environmental Health Sciences, School of Public Health University of Alberta, Edmonton, Alberta, Canada; 4 Department of Renewable Resources, University of Alberta, Edmonton, Alberta, Canada; University of Nevada, Reno, UNITED STATES OF AMERICA

## Abstract

Freshwater fishes are declining globally due to habitat loss, climate change, and other anthropogenic pressures. The Plains Sucker (*Pantosteus jordani*), recently recognised as a distinct species due to genetic and biogeographical factors, occurs east of the Rocky Mountains in Canada and the northern United States. Canadian populations are divided into the Missouri Basin and the Saskatchewan–Nelson Basin, but their genetic structure remains poorly understood. To assess population differentiation and connectivity, we analyzed mitochondrial DNA (7,770 bp) and eight microsatellite markers from 371 individuals across 21 sites in Alberta, Saskatchewan (Canada); and Montana (USA). Mitochondrial analyses revealed high haplotype diversity with significant regional clustering, particularly in the Frenchman River population, which was strongly differentiated from all other sites. Microsatellite analyses further supported basin-level structuring, with clear genetic separation between the Missouri and Saskatchewan–Nelson basins and strong differentiation among Missouri tributaries. In contrast, Saskatchewan–Nelson populations showed little genetic differentiation, consistent with recent or ongoing connectivity. The Frenchman River exhibited reduced genetic diversity, suggesting a history of drought-driven isolation and potential population bottlenecks. Together, these results highlight the influence of both historical colonization and contemporary fragmentation on the genetic structure of *P. jordani*. The Missouri Basin populations, with their restricted distributions and lower genetic diversity, may be particularly vulnerable to climate change and anthropogenic barriers, while Saskatchewan–Nelson populations appear more resilient but face ongoing threats from habitat alteration and pollution. By identifying distinct population units, this study provides critical insights for conservation planning and management of *P. jordani*.

## Introduction

Fish populations globally are declining due to both anthropogenic influences such as overharvesting, habitat loss and fragmentation, and pollution, as well as habitat changes due to climate change [1,2]. Freshwater populations have declined by as much as 80% over the past 50 years [2]. In North America, freshwater habitat has historically been shaped by a combination of intense tectonic/geologic activity and continent-wide climatic events such as the glaciations of the Pliocene and Pleistocene [3–5]. This physiography along with contemporary isolation has led to frequent speciation and extinction events, ultimately shaping not only the species distributions within river drainages but also the structure of populations [6].

The Catostomidae family consists of approximately 76 species of suckers within the order Cypriniformes that are found in freshwater habitats in North America [7]. The most speciose genus, *Catostomus*, is predominantly found in western North America where the western mountain ranges represent the primary barriers isolating species, leading to significant morphological variation both between and within species which has made the classification and resolution of the taxonomic status of this genus difficult [8,9]. Using fossil reconstruction and mitochondrial DNA sequences to examine the phylogeny of the genus, Unmack et al. [10] revealed significant differences among populations of several species of *Catostomus* and restored the genus *Pantosteus*, which was previously classified under *Catostomus*. As a result of the revised taxonomic classification, several new species were resurrected in western North America including the revision of *Pantosteus platyrhynchus* into the Mountain Sucker *P. platyrhynchus sensu stricto* (Cope, 1874) in the northern Great Basin, the Lahontan Sucker *P. lahontan* Rutter, 1903 in the Lahontan Basin of Nevada and California, the Cordilleran Sucker *P. bondi* (Smith, Stewart & Carpenter, 2013) west of the Rocky Mountains, and the Plains Sucker *P. jordani* Evermann, 1893 east of the Rocky Mountains [9,10].

**Fig 1:**
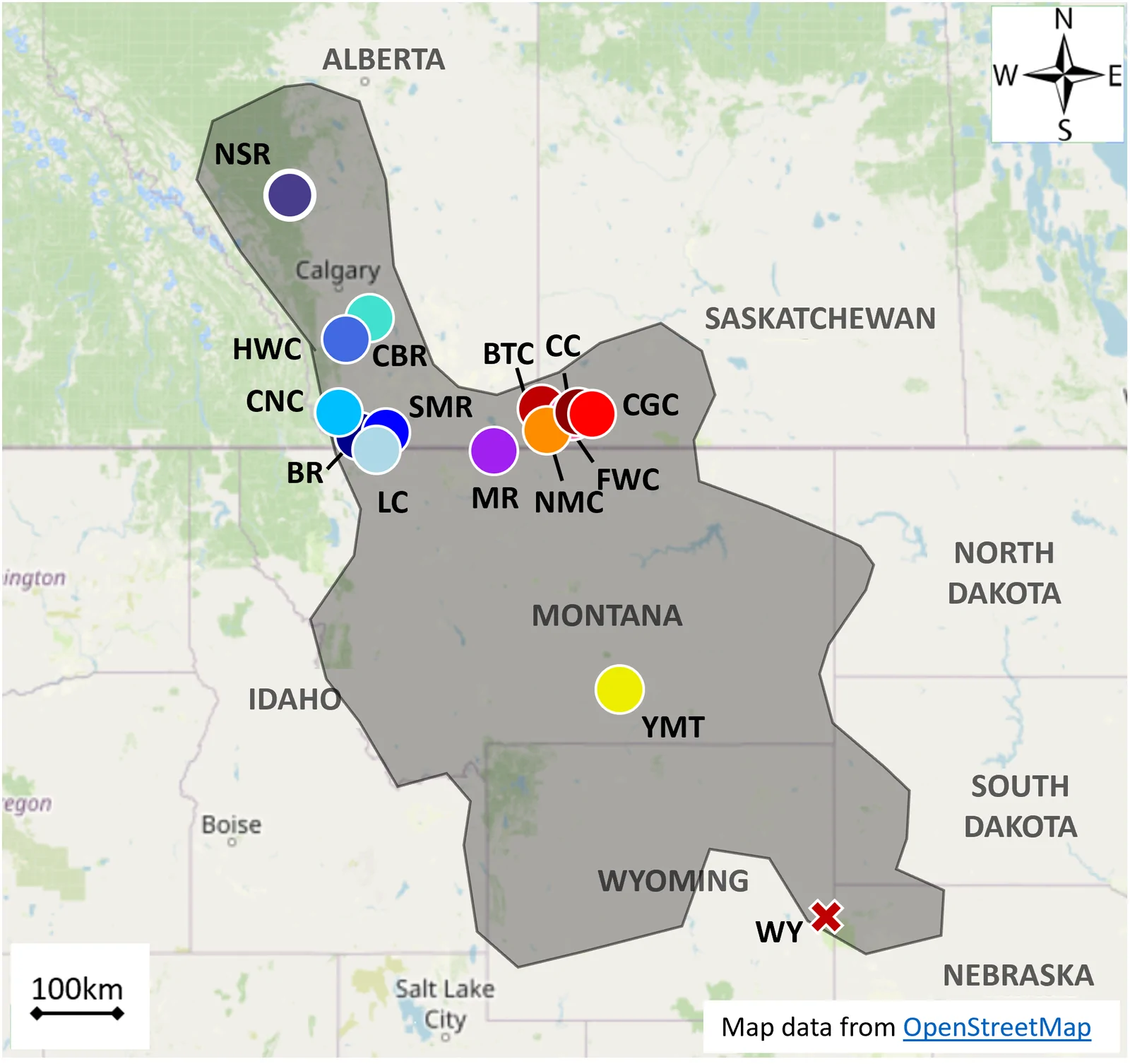
Approximate distribution (shaded) and sampling locations of Plains Sucker (*P. jordani*) from the Missouri Basin (red) and the Saskatchewan-Nelson Basin (blue). Distribution estimated from Boguski and Watkinson [11] and Unmack et al. [10]. Map was modified from OpenStreetMap [12]. Samples are colour-coded by sampling location (see Fig 2).

The Plains Sucker is found in Canada east of the Rocky Mountains in Alberta and Saskatchewan, as well as in parts of the northern United States including Montana and Wyoming [10]. The Plains Sucker is geographically divided into two main groups: the Missouri Basin, which includes the Milk River drainage of southern Alberta and Saskatchewan, as well as the Upper Missouri River of Montana, Wyoming, and South Dakota; and the Saskatchewan-Nelson Basin which has a more widespread distribution across the Saskatchewan River drainage, encompassing five tributaries in Alberta and Saskatchewan (Fig 1) [13,14]. In Canada the Plains Sucker Missouri populations have been listed as Threatened under the Species-at-Risk Act (SARA), while the Saskatchewan-Nelson populations are designated as Special Concern [13]. The taxonomic revision of *Catostomus* completed by Unmack et al. [10], only included specimens collected in US watersheds. Many of the basins where specimens were collected are shared between Canada and the US allowing for the results to be extrapolated to Canadian populations based on biogeography. However, the Saskatchewan River Basin was not considered in the analysis and population substructure may exist within the Canadian distribution given the limited contemporary connectivity between watersheds due to habitat (i.e., water temperature) or dams. As such, a comprehensive understanding of the population structure of Plains Suckers in Canada is crucial for the conservation and recovery of this species.

In this study we used a combination of mitochondrial DNA and microsatellites to explore the population genetic structure of *P. jordani* across much of its Canadian distribution. The study aims to identify whether there is genetic differentiation between the Missouri and Saskatchewan-Nelson Basin populations, whether there are any differences in genetic variation between basins, and whether there is evidence of ongoing gene flow both within and among regions.

## Materials and methods

### Sample collection

A total of 371 samples were collected from 21 sampling locations in seven regions found in two basins (Missouri and Saskatchewan-Nelson) from southern Alberta, southern Saskatchewan, and Montana (Fig 1, Table 1). The Milk River was sampled June-August of 2019 and 2020 and September-October of 2019. Fish were sampled using a beach seine net (6 m x1.2 m, mesh size 3 mm) and the entire width of the river was sampled for approximately 700–1000 m of the river. Due to the in-stream heterogeneity of the North Saskatchewan River (Saskatchewan Basin: Bow and Oldman Rivers) and Battle, Caton, Conglomerate Fairwell, Nine Mile creeks (Missouri basin), these systems were sampled using a Smith-Root™ LR-24 Backpack Electrofisher (Vancouver, WA, USA) using 275–350 V, 30 Hz, 15–20% (5.0–6.67 ms pulse width), 400 W (see [15]) during the June-August of 2020. Samples from Wyoming and Montana were provided upon request and incorporate a variety of sampling methods; Montana samples were collected in September 2015 by Montana Fish Wildlife and Parks; Wyoming samples were provided by the University of Wisconsin but were later removed as they were not *P. jordani*.

**Table 1:** Sample location, code, and sample size for 371 *P. jordani* samples from 21 locations grouped into seven regions according to basin.

Basin	Region	Location		Code	mtDNA (n)	msat (n)
Saskatchewan – Nelson Basin	North Saskatchewan River	North Saskatchewan River	AB	NSR	4	4
	Bow River	Carson Bow River	AB	CBR	5	10
		Highwood Creek	AB	HWC	5	31
	Oldman River	Connelly Creek	AB	CNC	5	9
		Belly River	AB	BR	4	29
		St. Mary’s River	AB	SMR	4	6
		Lee Creek	AB	LC	5	30
Missouri Basin	Milk River	Milk River (all)	AB	MR	5	130
		Milk River 1	AB	MR1		27
		Milk River 2	AB	MR2		21
		Milk River 3	AB	MR3		28
		Milk River 4	AB	MR4		25
		Milk River 5	AB	MR5		29
	Battle Creek	Battle Creek (all)	SK	BTC	5	33
		Battle Creek 1	SK	BTC1		7
		Battle Creek 2	SK	BTC2		4
		Battle Creek 3	SK	BTC3		15
		Battle Creek 4	SK	BTC4		7
		Nine Mile Creek	SK	NMC		5
	Frenchman River	Caton Creek	SK	CC	5	68
		Conglomerate Creek	SK	CGC	2	2
		Fairwell Creek	SK	FWC	2	3
	Yellowstone River	Yellowstone River	MT	YMT	5	11
**Total**					**56**	**371**

Regions include the North Saskatchewan River (NSR), Bow River (BW), and Oldman River (OL) in the Saskatchewan-Nelson basin, and the Milk River (MR), Battle Creek (BTC), Frenchman River (FM), and Yellowstone River (YR) in the Missouri basin. Location abbreviations include Alberta (AB), Saskatchewan (SK), and Montana (MT). Both the Milk River and Battle Creek sites consist of multiple sampling locations that were analysed both together and separately for the microsatellite analyses.

### Laboratory protocols

#### DNA extraction, amplification, and sequencing.

DNA from all individuals was extracted using Qiagen DNeasy 96 Blood & Tissue Kit (Qiagen, USA) following the manufacturer’s protocol.

Six mtDNA fragments were amplified for 56 individuals from 13 sampling locations (Table 1): ND1 (NADH dehydrogenase subunit 1), ND2, the ND4 region that contains ND4 and ND4L, a region that contained ND5 and ND6, cytochrome *b* (CYTB), and the ATPase coding region (ATP) that contains ATPase 6 and ATPase 8. Primer and temperature details are given in S1 Table. PCR products were examined on a 1% agarose gel and purified using Cytiva Sera-Mag Select beads following manufacturer’s instructions. Purified PCR products were sequenced with ABI Big Dye Terminator v 3.1 cycle sequencing reaction kit and sequenced in both the forward and reverse direction on an ABI 3730 DNA Analyzer. Note that ND4 and ND5/6 were both amplified and sequenced as two overlapping fragments. Additionally, CYTB and ND5/6 (2^nd^ fragment) used an internal sequencing primer to generate a 3^rd^ sequence as needed. The sequence chromatograms were aligned in Geneious (https://www.geneious.com) and per-individual consensus sequences were generated.

#### Microsatellite genotyping.

Three hundred and seventy-one samples were genotyped for eight microsatellite loci previously developed for catostomid species: CCAT35, CCAT44, Dlu409, Dlu4283, US3, US4, US9, and US10 (S2 Table) [16–18]. These samples included the 56 samples sequenced above and an additional 315 samples from a total of 21 sampling locations (Table 1). The new sampling locations included Nine Mile Creek in Saskatchewan as well as finer-scale sampling in Milk River (five sites) and Battle Creek (four sites). To promote adenylation during PCR amplification, GTT pigtails were added to reverse primers [19]. This reduced the potential for genotyping errors resulting from the occasional, non-templated addition of adenosine to the 3′end of PCR products. Loci were PCR amplified in three multiplexed reactions (S2 Table) and each reaction was then loaded into its own injection on an ABI 3730 DNA Analyzer. Individuals were genotyped using GeneMapper software and allele sizes were determined relative to the GeneScan 500 TAMARA size standard (Applied Biosystems).

### Genetic analyses

#### MtDNA.

Analyses were conducted on concatenated sequences of the six fragments. Sequences were visualized and aligned in MEGA11 (Molecular Evolutionary Genetics Analysis version 11) [20]. Given the continued taxonomic uncertainty in the *Pantosteus/Catostomus* species we began by aligning our samples to known *P. jordani* sequences, along with representatives of nine other *Pantosteus* species (*P. bondi*, *P. clarkii*, *P. discobolus*, *P. lahontan*, *P. nebuliferus*, *P. platyrhynchus*, *P. plebius*, *P. santaanae*, and *P. virescens*), seven *Catostomus* species (*C. ardens*, *C. catostomus, C. columbianus, C. commersonii, C. insignis, C. macrocheilus,* and *C. tahoensis*), and *Cycleptus elongatus* as an outgroup (S3 Table). A neighbor-joining tree was constructed in MEGA11 with 1,000 bootstrap replicates [21–23]. The molecular phylogeny of the *P. jordani* samples was repeated with maximum likelihood analyses in IQ-TREE 3 [24] with the *P. jordani, P. bondi,* and *P. lahontan* GenBank sequences along with *P. discobolus* as the outgroup. IQ-TREE 3 was run using ModelFinder [25] to select the best model based on the optimal BIC score and 10,000 replicates using UFBoot (ultrafast bootstrap approximation) [26] to determine branch support.

Haplotypes were assigned with TCS v1.21 [27] and confirmed manually. Variation was visualized with a statistical parsimony network constructed as a TCS network in PopART (Population Analysis with Reticulate Trees) [28,29] and with a principal coordinates analysis (PCoA) performed in GenAlEx v6.503 [30,31] using the haploid distance calculation. A statistical parsimony network was also run on each of the six fragments independently. Haplotype (H_d_) and nucleotide (π) diversity were calculated in DNAsp v5 [32]. Pairwise Φ_ST_ values were calculated in Arlequin v3.5 (p distances; 10,000 permutations) [33]; significance was determined after correction for multiple tests with a modified false discovery rate approach [34]. Isolation-by-distance was tested with a Mantel’s test performed in GenAlEx v6.503 on geographical distances determined with Google Earth as distance along the waterways in an “as the river flows” method and genetic distances were linearized Φ_ST_ values. Significance was tested with 1,000 permutations. Both a spatial analysis of molecular variance (SAMOVA) [35] and a series of hierarchical AMOVAs [36] run in Arlequin v3.5 were used to identify the optimal grouping within and between possible groups. To increase the population size, CGC and FWC were combined for all population analyses as they are both geographically adjacent and genetically similar

#### Microsatellites.

Samples missing genotypes for more than one locus (12.5% missing) were excluded from analyses. Data was checked for allelic dropout, null alleles, or slippage stutter with Micro-Checker v2.2.3 [37]. Linkage disequilibrium and deviations from Hardy Weinberg Equilibrium were tested with exact tests using Genepop v4.7.5 (1,000 batches, 1,000 iterations, 1,000 dememorisation steps) [38,39]. Per population observed (H_obs_) and expected (H_exp_) heterozygosity values were calculated in Arlequin v3.5 [33], and per locus values were calculated in R [40] with the *adegenet* 2.1.10 package [41,42]. Allelic richness with rarefaction to 8 alleles was calculated in HP-Rare v1.1 [43]. Population pairwise F_ST_ values were calculated in Arlequin v3.5 (100,000 permutations). Significance was corrected for multiple tests with the modified false discovery rate method [34]. A Mantel’s test was run in GenAlEx v6.503 [30,31] to test for isolation-by-distance with geographical distances calculated as above and linearized F_ST_ values [44]. Significance was tested with 1,000 permutations.

Clustering analyses were run in Structure v2.3.4 [45] to determine the number of genetic clusters (groups) present with no *a priori* population information. Structure was run with the admixture model and uncorrelated allele frequencies for 100,000 burn-in and 300,000 post burn-in MCMC steps for 10 runs each of K = 1–14. The results were averaged with Structure Harvester v0.6.6 [46] and the most likely number of clusters (K) was determined with both the Evanno ΔK method [47] and the highest penalized log likelihood method [45]. Runs were repeated with the two large samples, Milk River and Caton Creek, randomly reduced to 30 individuals to equalize sample sizes; results are similar and are not shown. Hierarchical clustering analyses were also run on the groups identified at K = 2 and on each river basin separately. A discriminant analyses of principal components (DAPC) was run in R [40] with the *adegenet* 2.1.10 package [41,42] to visualize the pattern of population genetic structure in the samples. The DAPC was run both on samples defined by sampling location (i.e., population) and on samples grouped into clusters identified with a k-means search and identified as the number of clusters with the minimum Bayesian Information Criterion (BIC) value.

## Results

### MtDNA

In total, 61 individuals from 14 sampling locations were sequenced across six fragments of mtDNA regions (GenBank accession numbers for the *P. jordani* samples: PX377551-PX377886): ND1 (915 bp), ND2 (1,029 bp), ND4 (1,521 bp), ND5/ND6 (2,357 bp), ATPase 6/8 (842 bp), and Cyt b (1,106 bp). The neighbor-joining tree clearly separated *Pantosteus* from *Catostomus* and identified 56 of the 61 samples as *P. jordani* (S1 Fig). Five samples collected from two locations on Rawhide Creek (42.464708, −104.375897; 42.354033, −104.329633) approximately 200 km north of Cheyenne, Wyoming (WY) grouped as *P. discolobus* and were subsequently removed from all further analyses. The maximum likelihood tree further supported the separation of the *P. jordani* samples from the recently separated *P. bondi* and *P. lahontan* samples while showing limited geographic clustering of the *P. jordani* samples with the exception of the CC and FWC samples which all grouped together (S2 Fig).

**Fig 2:**
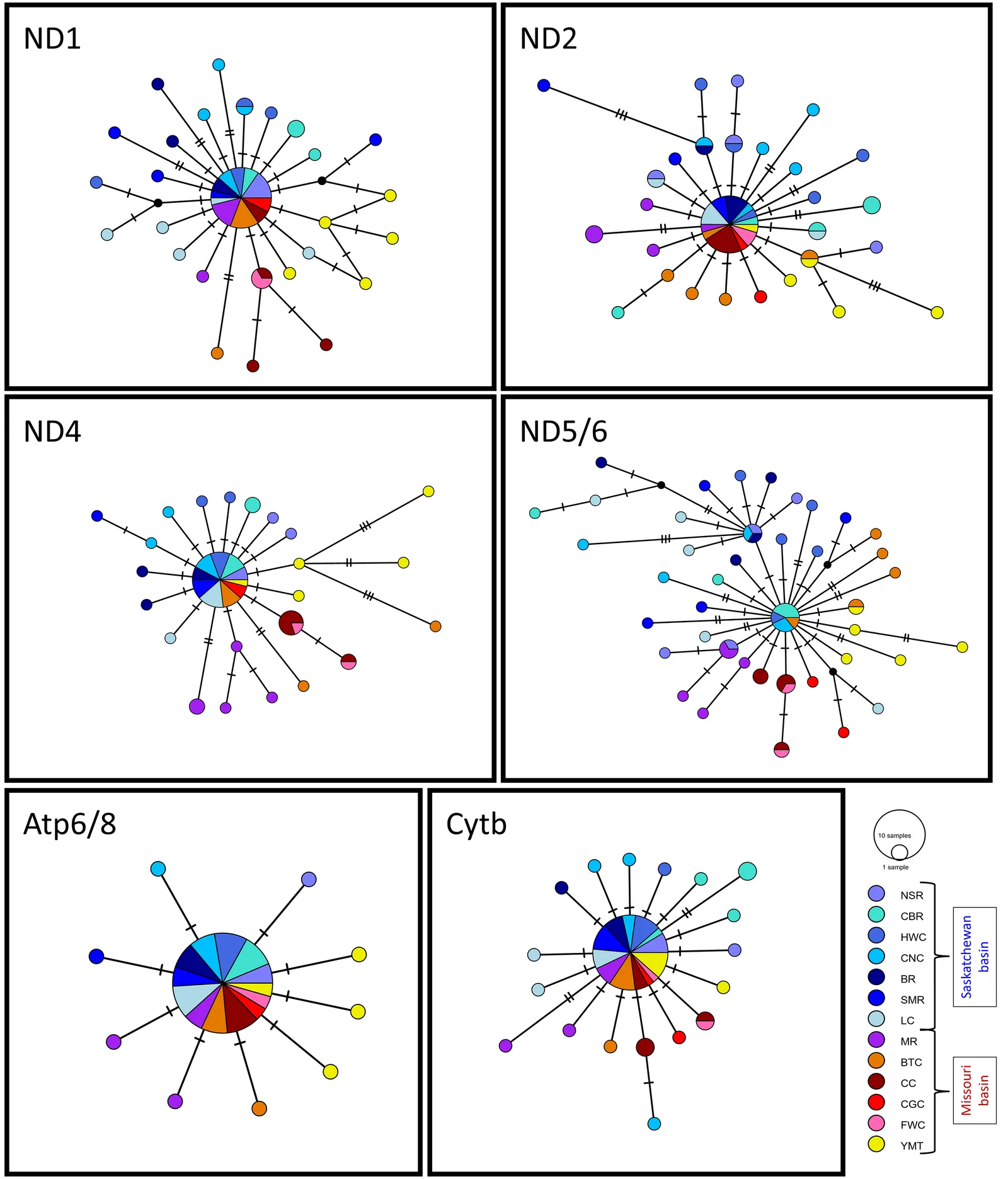
Statistical parsimony networks constructed on each of the six individual mtDNA fragments for 56 Plains Sucker samples from 13 locations. Each circle represents one or more samples, scaled for size, and each dashed line is a base pair change. Samples are colour-coded by sampling location (see Table 1).

The 7,770 bp concatenated sequence yielded 176 variable sites, 42 of which were parsimony informative. In the 56 individuals analyzed, 53 haplotypes were identified – three were shared between individuals from the same (for CBR and CC) or nearby (CC and FWC) sampling sites, and 50 were unique (Table 2; Fig 2). Haplotype diversity was consequently high in all populations (H_d_ = 0.9–1.0), and nucleotide diversity was also high across populations (Table 2), with the highest value observed in the Yellowstone River population (π = 0.0016) and the lowest in the Frenchman River populations (π = 0.0006–0.0008). The statistical parsimony network on the concatenated sequences revealed a pattern of clustering with haplotypes only shared within regions and most samples sharing their nearest neighbor within each region. Haplotypes differed by one to nineteen variable sites, with all samples radiating from a central unsampled haplotype (S3 Fig). The Yellowstone River samples were the most distinct both from the other samples and from each other. When the genes were analysed individually a similar pattern was evident with each gene showing a single central shared haplotype with samples radiating from the center in a starburst pattern (Fig 2). Interestingly, the CC and CGC samples did not have the central shared haplotype in the ND4 fragment, and none of the Frenchman River samples did in the ND5/6 fragment. Similarly, the Yellowstone River samples did not have the central shared haplotype in two of the fragments (ND1 and ND5/6) and were generally the most distinct. The ND5 network showed the greatest degree of structure with several Saskatchewan-Nelson River Basin samples forming a small cluster branching off of the shared central haplotype.

**Table 2:** Diversity values per population for 56 individuals sequenced at six concatenated mtDNA fragments and 371 individuals genotyped across 8 microsatellite loci.

	mtDNA					microsatellites				
Location	n	vs	h	H_d_	π	n	H_obs_	H_exp_	AR	PAR
North Saskatchewan River	4	13	4	1.000	0.0009	4	0.750	0.772	4.9	0.03
Carson Bow River	5	18	4	0.900	0.0011	10	0.800	0.876	5.6	0.31
Highwood Creek	5	19	5	1.000	0.0010	31	0.802	0.864	5.5	0.24
Connelly Creek	5	22	5	1.000	0.0012	9	0.833	0.851	5.2	0.42
St. Mary’s River	4	20	4	1.000	0.0013	6	0.896	0.839	5.4	0.18
Belly River	4	13	4	1.000	0.0008	29	0.815	0.850	5.4	0.29
Lee Creek	5	20	5	1.000	0.0011	30	0.791	0.859	5.5	0.20
Milk River (all)	5	19	5	1.000	0.0012	130	0.820	0.862	5.6	0.57
Milk River 1						27	0.822	0.872	5.8	0.23
Milk River 2	1					21	0.813	0.847	5.5	0.20
Milk River 3	4					28	0.811	0.869	5.6	0.22
Milk River 4						25	0.808	0.844	5.5	0.22
Milk River 5						29	0.844	0.870	5.6	0.23
Battle Creek (all)	5	21	5	1.000	0.0011	33	0.893	0.888	5.9	0.60
Battle Creek 1	1					7	0.911	0.891	6.0	0.25
Battle Creek 2						4	0.875	0.925	5.9	0.23
Battle Creek 3	3					15	0.933	0.884	5.8	0.22
Battle Creek 4	1					7	0.839	0.849	5.4	0.19
Nine Mile Creek						5	0.850	0.892	6.0	0.43
Caton Creek	5	9	4^b^	0.900	0.0006	68	0.734	0.829	5.0	0.24
Conglomerate Creek^a^	2	11	4^b^	1.000	0.0008	2	0.775	0.871	5.6	0.68
Fairwell Creek^a^	2					3				
Yellowstone River	5	29	5	1.000	0.0016	11	0.865	0.932	6.4	0.95
**Total**	**56**	**176**	**53**	**0.998**	**0.0012**	**371**				

Acronyms: sample size (n), number of variable sites (vs), number of haplotypes (h), haplotype diversity (H_d_), nucleotide diversity (π), per population observed (H_obs_) and expected (H_exp_) heterozygosity values, allelic richness (AR), and private allelic richness (PAR).

^a^CGC and FWC were combined for diversity measures due to sample size and proximity.

^b^CC and FWC share a haplotype.

Pairwise Φ_ST_ values (Table 3a) showed Caton Creek in the Frenchman River watershed to be significantly differentiated from all populations in the other six regions. The Milk and Yellowstone River samples also showed differentiation from other regions, although these differences were slight and not significant once corrected for multiple tests. When the samples were grouped into the seven tributaries (Table 3b), the Frenchman River samples were significantly differentiated from all other regions, and the Milk and Yellowstone River samples were significantly different from the Bow, Oldman, and Frenchman River samples, as well as from each other. The Battle Creek samples were not significantly different from any region other than the Frenchman River, and the three tributaries within the Saskatchewan-Nelson basin were not significantly different from each other. There was evidence of slight but significant isolation-by-distance among populations (p = 0.001).

**Table 3:** Population pairwise comparisons based on mtDNA (Φ_ST_, above diagonal) and microsatellites (F_ST_, below diagonal) for (a) sampling locations and (b) sites grouped into regions as in Table 1.

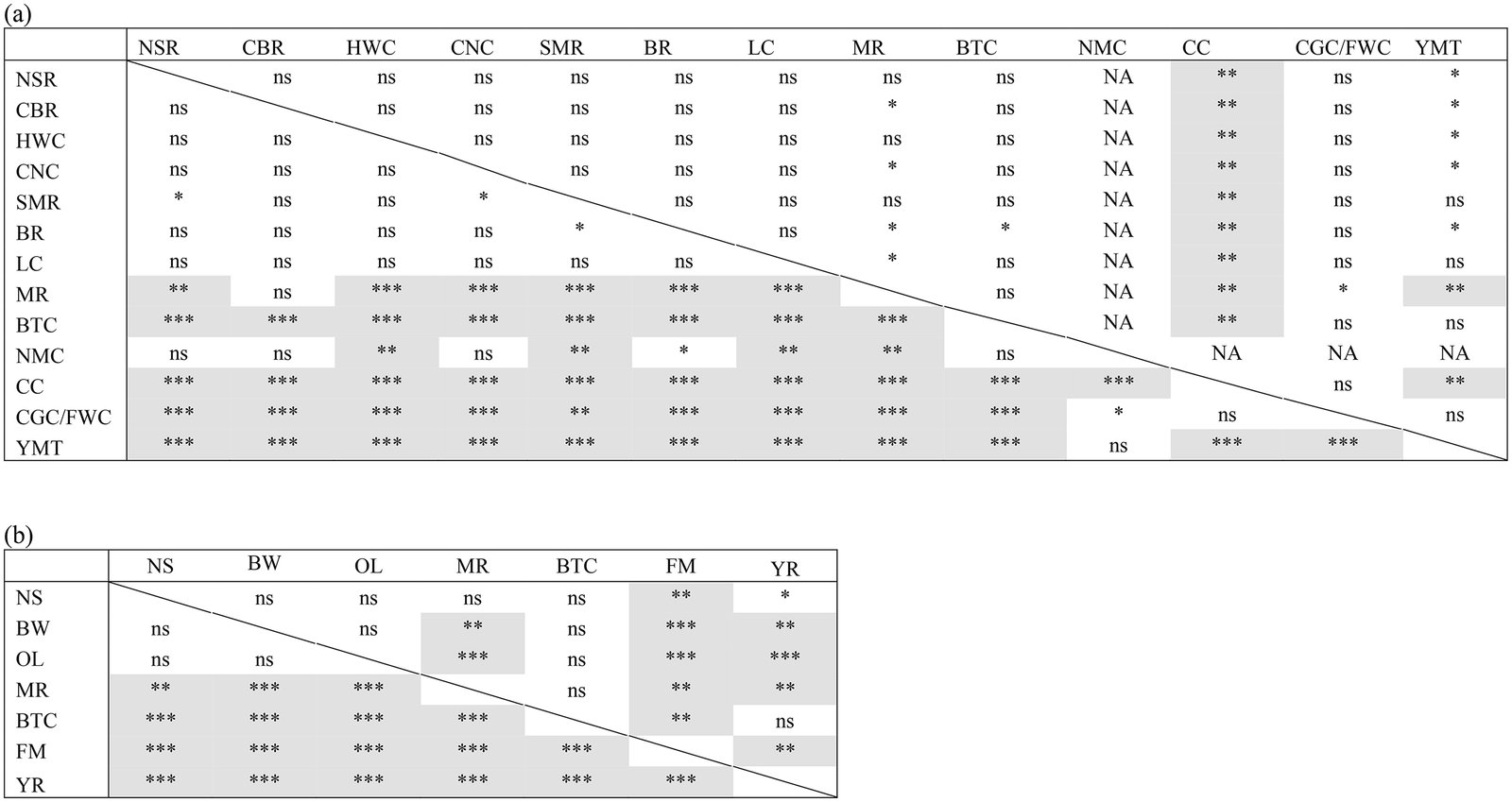

Significant values following correction for multiple tests are shaded; P_crit_ = (a) 0.010 and (b) 0.014. *** p < 0.001, ** p < 0.01, * p < 0.05, ns p ≥ 0.05.

**Fig 3:**
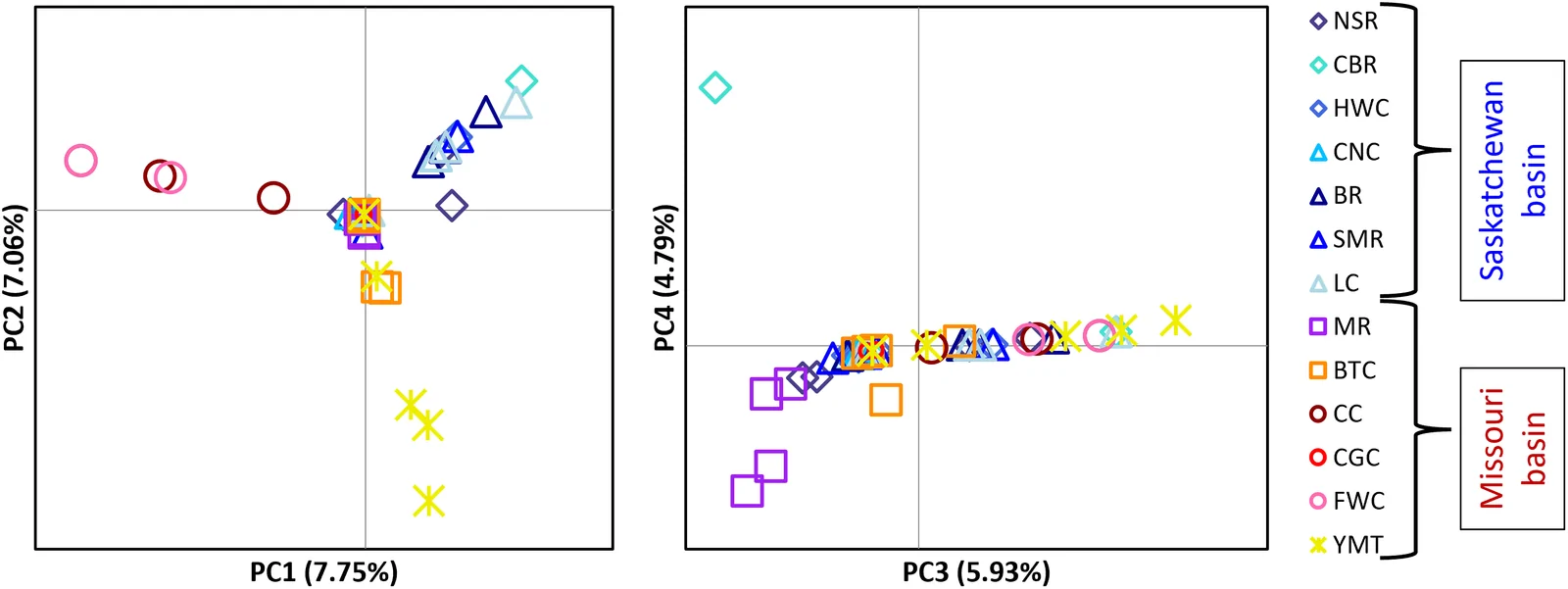
Principal coordinates analysis (PCoA) on concatenated mtDNA sequences from 56 Plains Sucker samples from 13 sampling locations. Samples are colour-coded by sampling location and shapes correspond to the six regions as in Table 1.

The SAMOVA identified four groups based on genetic and geographic data (Φ_CT_ = 0.158, p < 0.001): the Frenchman River (CC, CGC, FWC), Yellowstone River (YMT), Milk River (MR), and Battle Creek combined with the Saskatchewan basin samples (BTC, NSR, CBR, HWC, CNC, BR, SMR, LC). In both the SAMOVA and the hierarchical AMOVAs, as the number of groups was increased from K = 2 we saw the same pattern: the Yellowstone River samples formed the first separated group, followed by the Frenchman River samples, and then the Milk River samples; Battle Creek was not identified as a group until K = 6. When the samples were run as the seven regions defined in Table 1 the among-group variance was slightly lower than optimal but remained strongly significant (Φ_CT_ = 0.126, p < 0.001), while the rest of the variation was explained by the within-population differences (Φ_ST_ = 0.116, p < 0.001; Φ_SC_ = 0.000; p = 0.316). The PCoA (Fig 3) supports this pattern, with the Frenchman River (CC, CGC, and FWC) separating along coordinate 1 (7.8% of the variation), the Yellowstone River separating along coordinate 2 (7.1%), and the Milk River separating along coordinate 3 (5.9%). While the Battle Creek samples generally clustered together, there was extensive overlap with the Saskatchewan-Nelson Basin samples.

### Microsatellites

A total of 371 individuals from 21 sampling sites were genotyped for at least seven of the eight microsatellite loci. MicroChecker v2.2.3 did not detect any null alleles, allelic dropout, or input errors. Exact tests identified departures from Hardy-Weinberg equilibrium in five of the eight loci after correction for multiple tests (p_crit_ = 0.01): CCAT35 (CC), CCAT44 (MR, YMT), Dlu409 (CC), Dlu4283 (CBR, HWC, LC, CNC, CC, MR), and US3 (HWC). For all but Dlu4283 the deviation was restricted to one or two populations. Exact tests for linkage disequilibrium showed only six out of 364 significant deviations after correction for multiple tests (P_crit_ = 0.008); five of these were in the Milk River population, and the only locus-pair to appear twice was US3 and Dlu4283 which was significant in both HWC and MR. To ensure robustness of results, analyses were repeated with Dlu4283 removed; results are similar and not shown.

Observed heterozygosity was slightly lower than expected heterozygosity in 17 of the 20 comparisons and in all of the eight loci (Table 2), although the overall difference was not significant (Bartlett’s K-squared = 1.36, p = 0.24). At the population level, only the Yellowstone River population showed a significant difference between observed and expected heterozygosity (Bartlett’s K-squared = 21.4, p = 3.8 x 10^−6^). Overall, the greatest differences were evident in the Frenchman River populations (CC and CGC/FWC), while SMR and two of the BTC sites showed higher H_obs_ than H_exp_. On a per locus basis the greatest difference between H_obs_ and H_exp_ was seen in Dlu4283 followed by CCAT44 (S4 Table). Allelic richness values were similar across populations (4.9–6.4; average = 5.5) with the highest values seen in the Yellowstone River and Battle Creek and the lowest in Caton Creek and the North Saskatchewan River (Table 2). Private allelic richness varied considerably across sites (average = 0.298 with MR and BTC individual sites or 0.395 with MR and BTC subsites combined together) with the highest diversity in YMT (0.95) and the lowest in NSR (0.03; Table 2).

**Fig 4:**
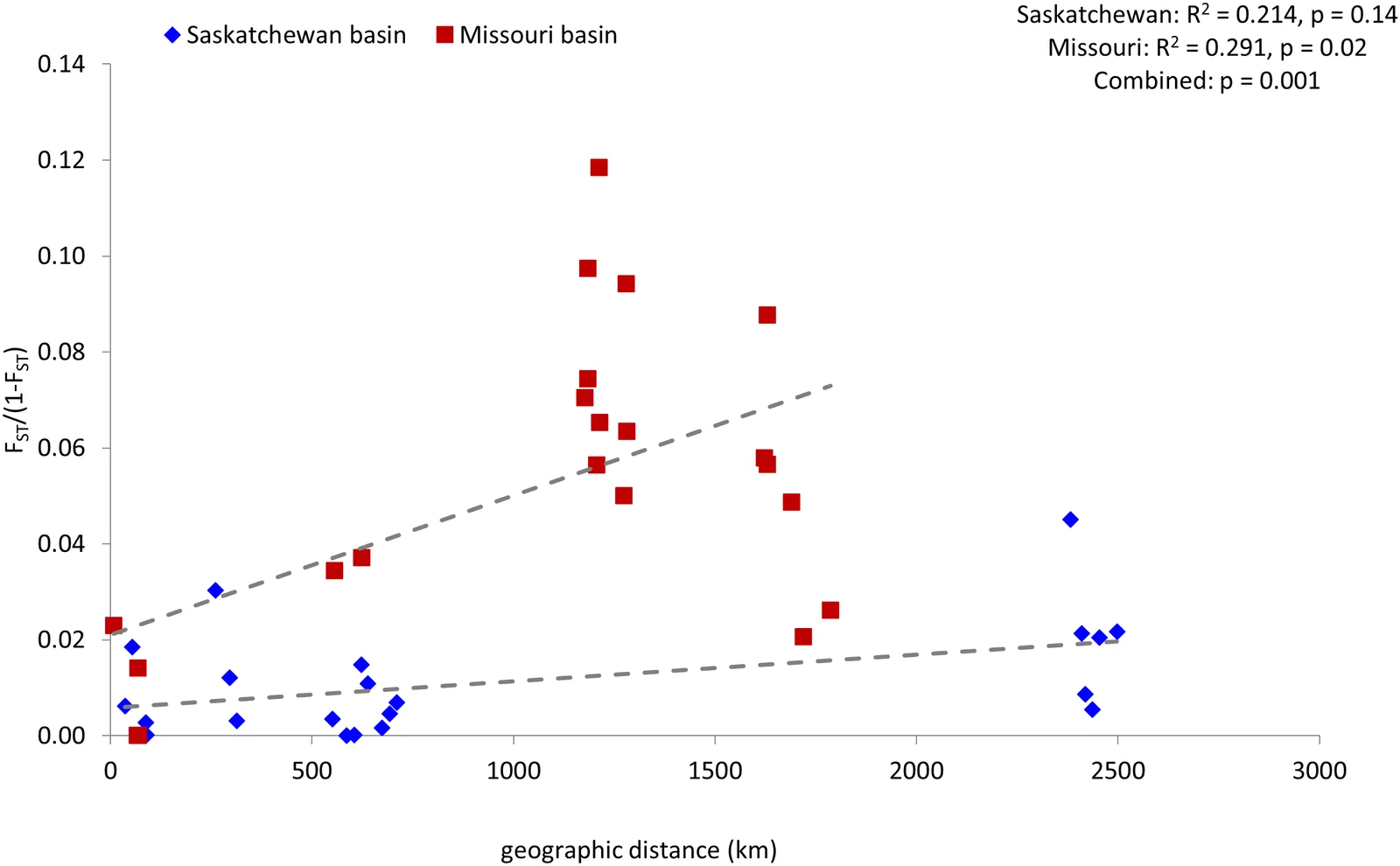
Isolation by distance analysis for 371 Plains Sucker samples genotyped at eight microsatellite loci tested both within basin and combined.

Global F_ST_, based on the eight loci, showed highly significant structuring among populations (F_ST_ = 0.05, p < 0.001). Population pairwise F_ST_ values were significant in 48 of the 78 comparisons (Table 3a); sites within the Saskatchewan-Nelson Basin were not significantly differentiated from each other, while those within the Missouri Basin (11 of 15 comparisons) or between basins (37 of 42 comparisons) were significantly different following correction for multiple tests (P_crit_ = 0.01). This was even clearer when comparisons were repeated on the populations grouped as the seven regions (Table 3b); regions within the Saskatchewan-Nelson basin were not significantly different while all other comparisons were. There was evidence of significant isolation-by-distance when all populations were tested (between basin distances were set at 10,000 km; p = 0.001), and in the Missouri Basin when the two basins were tested individually (R^2^ = 0.291; p = 0.02; Fig 4). The populations in the Saskatchewan-Nelson Basin showed a similar pattern (R^2^ = 0.214; p = 0.14), although the low genetic differences despite large geographical distances between NSR and the other populations likely caused the lack of significance. In both cases geographic distances between regions may have been overestimated as there may exist either historical or ephemeral connections between the larger river systems that were overlooked.

**Fig 5:**
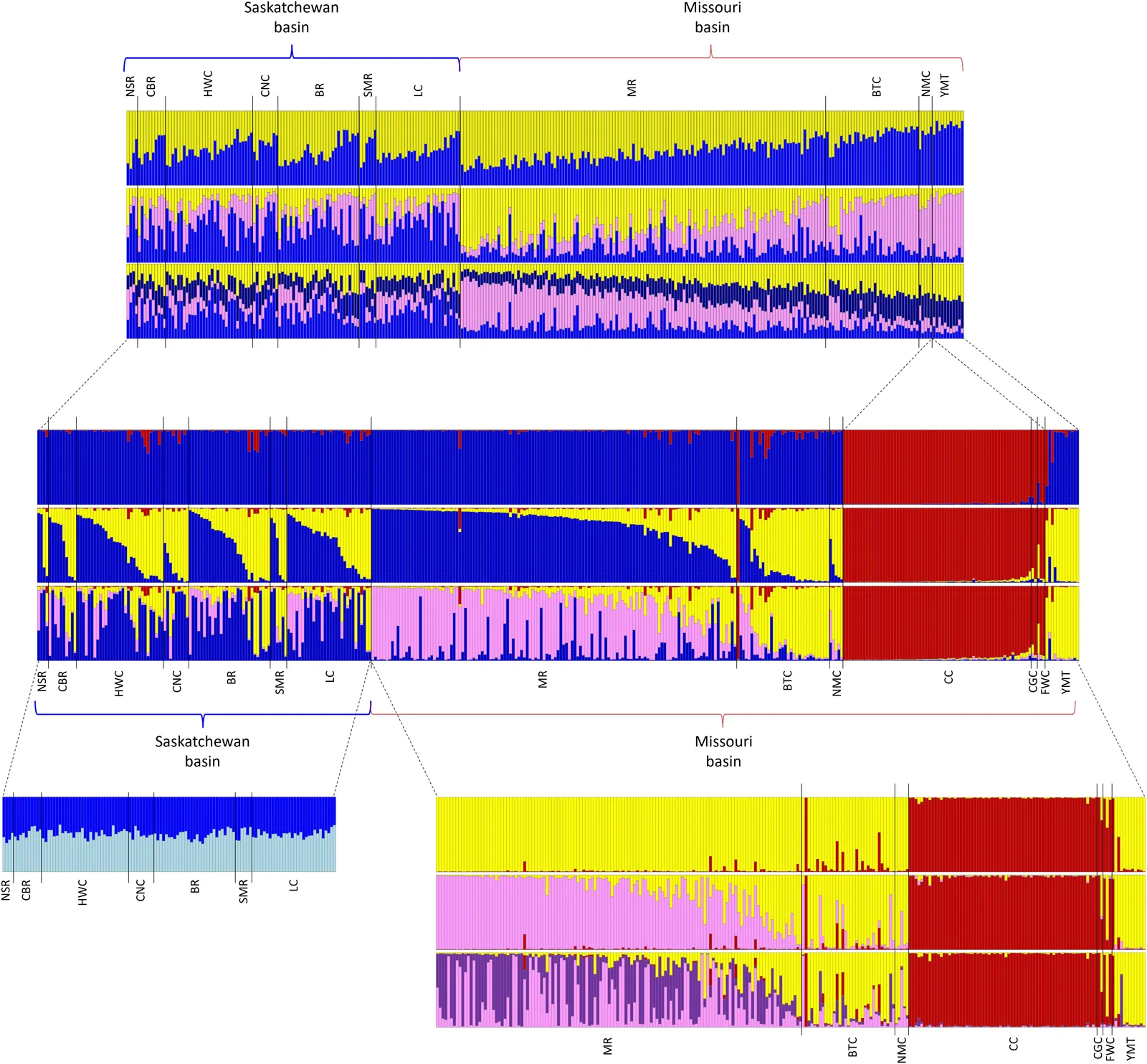
Bayesian clustering analyses of 371 Plains Suckers from 14 sampling locations genotyped at eight microsatellite loci for (a) K = 2, (b) K = 3, and (c) K = 4. Within each sampling location samples are ordered based on cluster membership at K = 3. Hierarchical analyses are shown for (top) the Frenchman River samples removed and (bottom) the Saskatchewan-Nelson basin (left) and Missouri basin (right) samples.

**Fig 6:**
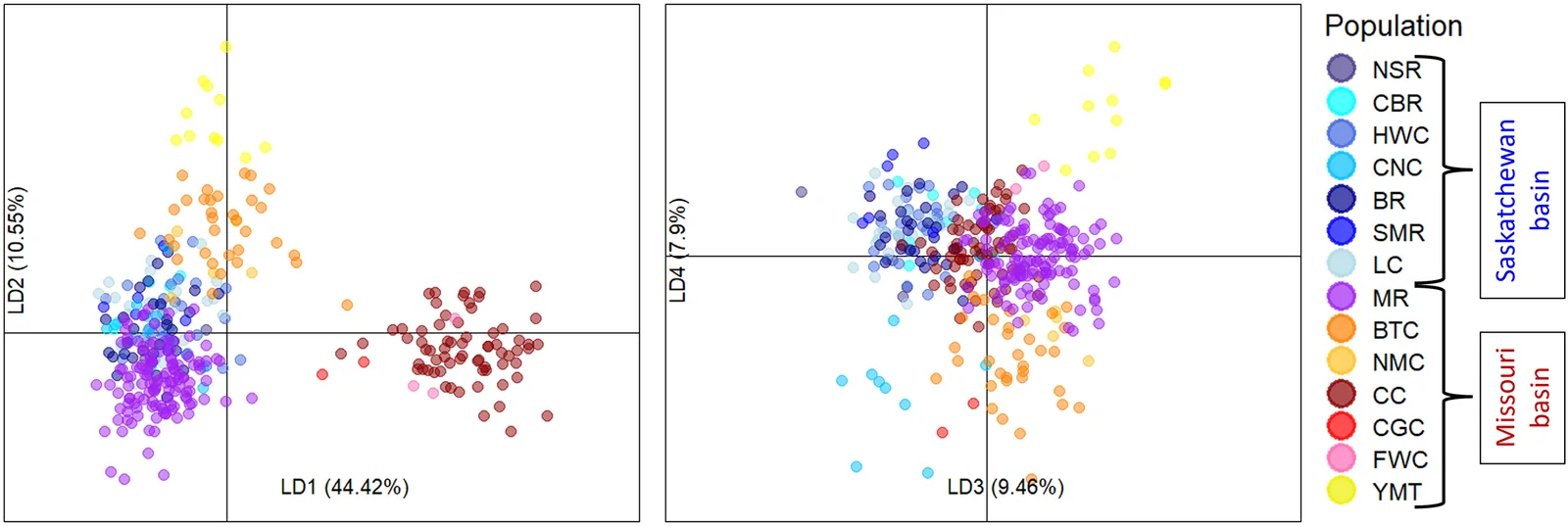
Discriminant analysis of principal components (DAPC) on 371 Plains Suckers from 14 sampling locations genotyped at eight microsatellite loci.

Bayesian clustering analyses identified two groups with Evanno’s ΔK: the Frenchman River and all other populations, while log likelihood penalized tests supported four groups (Bayes factor = 1.0, ln Pr (X | K) = −15,225): the Frenchman River, Milk River, Yellowstone River with Battle Creek, and the Saskatchewan basin populations, although there was notable admixture in the latter three groups (Fig 5). At higher values of K, levels of admixture increased, primarily in the Battle Creek and Yellowstone River populations, while the Frenchman River sites remained strongly differentiated. When run without the Frenchman River samples two groups were identified with Evanno’s ΔK, the Saskatchewan-Nelson Basin and the Missouri Basin, while log likelihood penalized tests supported three groups (Bayes factor = 1.0, ln Pr (X | K) = −9784) with the additional separation of the Milk River populations, albeit with extensive admixture in all groups (Fig 5). A similar pattern was evident when the two basins were run separately: the Missouri populations separated as the Frenchman River, Milk River, and Battle Creek with Yellowstone River, whereas no additional substructuring was seen in the Saskatchewan-Nelson populations (Fig 5). Similarly, the DAPC (Fig 6) showed clear separation of the Frenchman River populations along LD1 (44.4% of the variation), with separation of MR, BTC, and YMT from the other sites along LD2 (10.6%) and LD3 (9.5%). LD4 (7.9%) again differentiated the BTC and YMT samples from the other sites and also separated the CNC samples from the other sites in the Saskatchewan-Nelson Basin, the only evidence of population structure in the Saskatchewan-Nelson Basin. When the DAPC was run on clusters identified from the k-means search the minimum BIC value suggested five clusters as the optimal value. These clusters represented one that contained all but one Frenchman River sample, and four clusters that contained samples from all other populations. The Frenchman River cluster separated along LD1 which explained 57.6% of the total variation. The other four clusters separated slightly along LD2 which explained 18.2% of the variation.

## Discussion

While there is considerable knowledge of the morphology and biology of Plains Sucker [9,48], little is known about the population genetic structure of this species in Canada. When the current taxonomy for Plains Sucker was established, only two *P. jordani* samples were included, both from the Missouri River Basin in the United States (WY and SD) [10]. While these samples clearly supported the separation of *P. jordani* as a distinct species, they were unable to fully encompass the genetic diversity and differentiation present in the species. The current study extends previous genetic analyses for this species by providing the first documentation to describe the genetic diversity and population structure of Plains Sucker in Canada, including samples from both the Saskatchewan-Nelson and Missouri River basins. Results from six mitochondrial (mt) DNA fragments and eight nuclear microsatellite loci showed significant variation among populations, most notably between the two river basins (Missouri and Saskatchewan-Nelson) and among the Missouri River Basin tributaries. Interestingly, the Frenchman River samples stood out as distinct from all other populations.

Both morphological and genetic differences between river drainages have previously been noted in many Catostomidae species [10]. For example, populations of the congeneric Bluehead Sucker (*Pantosteus discobolus*) in Bonneville Basin and the Upper Snake River are clearly distinct from those in the Colorado River Basin, with additional structure evident among Colorado River Basin populations [49]. A previous report on what was formerly called the Mountain Sucker (*Pantosteus platyrhynchus sensu lato*) also showed differences between river basins with four distinct groups identified using mtDNA: two clades from the (now) *P. jordani* from the Upper Saskatchewan and Missouri drainages in Canada, one clade, presumably *Pantosteus bondi*, from the Columbia/Fraser drainages, and one clade, presumably *P. platyrhynchus sensu stricto*, from the Upper Snake River in Idaho [50]. In the current study, genetic differentiation was evident between the Missouri Basin and Saskatchewan-Nelson Basin, albeit with overlap of genotypes and shared alleles between the Milk River, Battle Creek, and Saskatchewan-Nelson River Basin populations. The sharing of alleles may be due to a relatively recent common ancestry between the regions as ongoing genetic exchange between the two basins is unlikely given that they have been physically isolated since the end of the Pleistocene glaciations [4]. The two drainages also exhibit distinct physical and ecological characteristics such as differences in water temperature and community structure that may have further differentiated the populations while maintaining selectively neutral similarities [14]. These distinct characteristics may have contributed to the vastly different genetic structure seen in the two different basins.

While our results support the genetic distinction between the Saskatchewan River and Missouri River basins, we also identified strong differentiation among samples within the Missouri River between the Frenchman River, Milk River, Battle Creek, and Yellowstone River populations. Although this separation is evident with both mitochondrial and nuclear markers, the pattern is much clearer with the microsatellite markers suggesting that it may be caused by a reduction or inhibition of contemporary gene flow rather than historical isolation alone. The populations within the Missouri Basin have been physically separated by such manmade structures such as the Fresno Dam in Havre, MT (built in 1939) and the Eastend Reservoir in Saskatchewan (built in 1936) (although see also: https://gis.wsask.ca/WSAOwnedDams/) for less than 100 years. Even recent fragmentation by manmade structures can affect the genetic structure of fish species, likely due to the complete cessation of gene flow in the absence of human assistance, coupled with the smaller resulting population sizes. For example, the Blue Sucker (*Cycleptus elongatus*) shows evidence of within-basin population structure in the Missouri River following construction of dams between 1952 and 1963, whereas the unimpounded Mississippi River does not show the same level of fragmentation [51]. In addition, the lack of records of Plains Suckers in the lower reaches of the Milk River, Battle Creek, and Frenchman River suggest that ecological barriers such as intolerable water temperature resulting in unsuitable habitat have likely isolated the populations in these tributaries from the rest of the Missouri River drainage, and from each other, for millennia [14]. These results are consistent with those found in *P. jordani* in the Black Hills of South Dakota at the south-eastern extent of their range where fish in the neighboring Belle Fourche River and Cheyenne River tributaries were found to be genetically distinct from each other despite geographic proximity and a lack of physical barriers [52].

Given the relatively short isolation of the Plains Sucker populations in Canada, the magnitude of genetic differentiation between the Frenchman River populations and the rest of the Missouri Basin populations was unexpected. The Frenchman River, a crucial wildlife habitat corridor, begins in the southwestern part of Saskatchewan, east of Cypress Hills, and flows south-east through Grasslands National Park (SK) before flowing into the Milk River and subsequently into the Missouri River in Montana. The Frenchman River is located on the southern fringes of the renowned Palliser Triangle, which represents one of the driest areas within the Canadian prairies [53]. A series of significant droughts, particularly in the prairie regions, have been recorded spanning various periods between 1910 and 2015 [54–56]. More recently, Montana, the Dakotas, and the Canadian Prairies were impacted by a severe drought during the spring and summer of 2017, significant due to its swift onset and intense escalation [57]. The Missouri River Basin, which encompasses the Frenchman River, was among the areas affected by this and previous droughts, with Saskatchewan bearing the brunt of the severe impacts and the widest extent of the drought. Severe drought conditions have been shown to lead to fragmentation and reduced gene flow between regions [58,59]. Fisheries and Ocean Canada has sampled Caton and Fairwell creeks in August and September of 2003, 2004, 2017, 2018, and 2023; across all years Caton Creek has had flow, but Fairwell Creek had only had isolated pools and downstream of the confluence of Caton and Fairwell creeks there was typically no surface flow or connectivity to the Frenchman River. Given the prevalence and severity of present-day droughts in this area, it is likely that the Frenchman River may have experienced similar conditions in the past, and likely will continue to do so in the future, resulting in both ongoing and worsening isolation and declining population trends. If the populations did undergo a reduction in population size as a result of this fragmentation, whether gradually or as a population bottleneck, then the effect of isolation and related genetic drift would have been accelerated relative to larger, more stable populations. This is supported by the lower genetic diversity in the Frenchman populations relative to the other Missouri Basin populations, as well as the lower observed than expected heterozygosity which can suggest a small population or population bottleneck [60].

The within-basin structure also highlighted the Yellowstone River population as different from the Canadian Missouri Basin populations. Given the current distribution of the Catostomidae, it has been suggested that *P. jordani* originated in the Missouri Basin and colonized northwards, likely following retreat of the glaciers [10]. A recent evolutionary history could explain the starburst pattern seen with the mitochondrial sequences, as has been found in both the Small Yellow Croaker (*Larimichthys polyactis*) and the Six Bar Wrasse (*Thallasoma hardwicki*) in the western Pacific Ocean [61,62]. Similarly, a southern origin is supported both by the relatively high heterozygosity, allelic richness, and distinct haplotypes observed in the Yellowstone River population, as well as by the low genetic diversity, paucity of private alleles, and lack of genetic structure found in the more northern and peripheral Saskatchewan-Nelson Basin populations. Decreasing diversity with increasing distance from a source population such as a glacial refugium is commonly seen due to founder effects and population bottlenecks; for example, the Broad Whitefish (*Coregonus nasus*) showed a loss of genetic diversity with post-glacial colonization from a northern Beringian refugium [63], while the Longnose Sucker (*Catostomus catostomus*) showed signs of decreased diversity in previously glaciated regions in Labrador, Canada [64]. Subsequent isolation as river drainages changed and connectivity decreased would then have led to significant population isolation and structure.

The evolution of Plains Sucker populations in Canada has been affected by a number of historical and contemporary barriers which may limit the movement and viability of populations, leading to increasing differentiation between regions. While populations in the two basins are all facing population decline from habitat fragmentation and destruction, the principal causes differ with the Saskatchewan-Nelson Basin populations facing increased risk from pollution due to wastewater runoff and effluent, whereas the Missouri Basin populations are at greater risk from climate change and severe weather affecting both water quality and quantity [14,48]. In addition, the Canadian populations in the Missouri Basin have a limited distribution where they are restricted to the Milk River drainage of southern Alberta and Saskatchewan [48]. As such, these populations are more susceptible to habitat loss and degradation from altered flow regimes and so will see a greater impact from genetic drift as differentiation is reinforced in smaller and more isolated populations where there is no influx of new alleles to homogenise populations or to replace lost diversity, and alleles tend to be lost at a higher rate when population size is small. On the other hand, despite major changes to the freshwater habitat due to increased urbanization and development such as the construction of the Oldman River Dam [48,65], Plains Sucker populations in the Saskatchewan-Nelson River Basin remain widespread with little genetic differentiation observed among tributaries. This lack of genetic structure further supports the notion of a southern origin to the Canadian populations, with a later colonization into the Saskatchewan-Nelson Basin, and suggests ongoing or recently ceased gene flow among tributaries. This contrasts with a previous study on a disjunct peripheral population of Mountain Sucker in the Black Hills, South Dakota, USA where habitat fragmentation caused by human activity resulted in reduced gene flow among streams [52].

Limited connectivity due to both historical and contemporary barriers, and subsequent genetic drift in the absence of gene flow, likely act in concert to shape the genetic structure of Plains Sucker in Canada. Ongoing threats to the species involve the degradation of habitat from a combination of climatic and anthropogenic causes – identified threats include climate change leading to severe weather, changes to water temperature, and severe droughts, loss of habitat from the construction of dams and reservoirs, pollution due wastewater and industrial effluent, and the introduction of invasive species [14]. By elucidating the population genetic structure in this species, this study provides new insights into potential conservation and management issues.

## Supporting information

S1 Table Primer sequences, source, and annealing temperature (T_A_) for amplification of six mtDNA fragments. (DOCX)

S2 Table Primer sequences, multiplex combination, final primer concentration (Conc (μM)), allele size range, and primer source for eight microsatellite loci. (DOCX)

S3 Table GenBank accession numbers for the catostomid sequences used in the phylogenetic analyses. (DOCX)

S4 Table Per locus observed (H_obs_) and expected (H_exp_) heterozygosity values for 371 plains sucker samples genotyped at eight microsatellite loci. A * denotes a significant difference between H_obs_ and H_exp_ after correction for multiple tests (p_crit_ = 0.0096).
(DOCX)

S1 Fig Neighbor-joining tree showing the relationship between the 61 samples sequenced, ten *Pantosteus* species, and seven *Catostomus* species, rooted to the confamilial *Cycleptus elongatus.* Alignment includes six mtDNA fragments totalling 7,770 bp. Bootstrap values are given for values above 80 and genera are highlighted by solid lines.
(TIF)

S2 Fig Maximum likelihood tree run in IQTree3 showing the relationship between the *56 P. jordani* samples sequenced, *Pantosteus jordani, P. lahontan,* and *P. bondi*, rooted to *P. discobolus.* Alignment includes six mtDNA fragments totalling 7,770 bp. Bootstrap values are given for major branches (1000 bootstrap iterations), and sequenced samples are colour-coded by location as in Fig 2.
(TIF)

S3 Fig Statistical parsimony network constructed with six concatenated mtDNA sequences totalling 7,700 bp from 56 Plains Sucker samples from 13 locations. Each circle represents one or more samples, scaled for size, and each dashed line is a base pair change. Samples are colour-coded by sampling location (see Table 1).
(TIF)
