# Supplementary material for: Population structure of Plains Sucker Pantosteus jordani (Evermann 1893) in Canada
Source: PLoS One. 2026 Aug 26;21(8):e0355547. doi: 10.1371/journal.pone.0355547 (PMC13514028; doi:10.1371/journal.pone.0355547)
Supplement: Supplementary file 1 [file pone.0355547.s001.docx]

**S1 Table.** Primer sequences, source, and annealing temperature (T_A_) for amplification of six mtDNA fragments.

| **mtDNA region** | **Primer Name** | **Primer Sequence** | **Primer Source** | **T_A_ (^o^C)** |
| --- | --- | --- | --- | --- |
| ATPase 6/8 | Lys.22F | AAAGCGTTAGCCTTTTAAGC | [10] | 48 |
|  | CO3.Cato.23R | GGCTTGGATCAACCATATGAT | [10] |  |
| CytB | Glu31 | TGRCTTGAAAAACCACCGTTGT | [10] | 52 |
|  | Pan.Thr.52 | GCTCTAGGGAGGAGTTTAACC | [10] |  |
| CytB (internal) | Pan.494F | GAGGTGGATTTTCAGTAGATAATGC | [10] | - |
|  | Pan.636R | AAGAGATTTTGTCCGCATCCGAG | [10] |  |
| ND1 | Leu3F | TAAGGTGGCAGAGCATGGTAAT | [10] | 48 |
|  | Ile24R | CTCTATCAAAGTGGTCCTTGG | [10] |  |
| ND2 | ND2.B2 | CTCCTGGTGCTTCCTCTACA | [10] | 45 |
|  | ND2.EgilaF | CTGCTTAGAGCTTTGAAGGC | [10] |  |
| ND4 (part 1) | Cato.Arg.2F | CAAGACCTCTGATTTCGGCTC | [10] | 58 |
|  | Cato.ND4.598R | TTACGAGGAAGGCAATTAAGCA | [10] |  |
| ND4 (part 2) | Cato.ND4.369F | GAAGCCACTCTAATTCCAACCTTA | [10] | 48 |
|  | Cato.His.R | CACAATCTAGTGTTTTAAGTTAAAC | [10] |  |
| ND5/6 (part 1) | LEU.1F | GTCTTAGGAACCAAAAACTCTTG | [10] | 55 |
|  | Cato.ND5.907R | CTGGATGTAGAGAAGGCTACAAT | [10] |  |
| ND5/6 (part 2) | Cat.ND5.739F | GCCCTACTCCACTCAAGCAC | [10] | 60 |
|  | Cato.GLU.2R | TAGTTGAATAACAACGGTGGTTC | [10] |  |
| ND5/6 (part 2 – internal) | Cato.ND5.1636R | GGACAAACAATCGCCACCCA | [10] | - |
|  |  |  |  |  |
