# Supplementary material for: Population structure of Plains Sucker Pantosteus jordani (Evermann 1893) in Canada
Source: PLoS One. 2026 Aug 26;21(8):e0355547. doi: 10.1371/journal.pone.0355547 (PMC13514028; doi:10.1371/journal.pone.0355547)
Supplement: Supplementary file 2 [file pone.0355547.s002.docx]

**S2 Table.** Primer sequences, multiplex combination, final primer concentration (Conc (**μ**M)), allele size range, and primer source for eight microsatellite loci.

| **Locus Name** | **Primer Sequence** | **Multiplex** | **Conc (μM)** | **Allele range** | **Source** |
| --- | --- | --- | --- | --- | --- |
| CCAT35 | /5TET/AGGCATCAAATCCTTGGCAG | MS1 | 0.1 | 192-252 | [16] |
|  | GTTCCTGTGAGACTGTGTGAAACC |  |  |  |  |
| CCAT44 | /56-FAM/CATCGGAATGGCTTCATGGG | MS1 | 0.8 | 311-341 | [16] |
|  | GTTAAATTAAATGAGCCTGAGGTGG |  |  |  |  |
| DLU409 | /5HEX/TGCGATCCTAGAAGGAGTAAAACA | MS1 | 0.4 | 225-313 | [17] |
|  | GTTATTCCATTTGCTGTCAACTTCAAA |  |  |  |  |
| DLU4283 | /5TET/TGGTTGGATGCTGAAATAAAGTAA | MS3 | 0.2 | 184-300 | [17] |
|  | GTTGAGCCTCATCATAGATGGATAGA |  |  |  |  |
| US3 | /5HEX/CCCTGGGAGCATCAGTTAGA | MS3 | 0.1 | 97-137 | [18] |
|  | GTTAAAGGTTGTGACCCACTGC |  |  |  |  |
| US4 | /56-FAM/GCATGCTGTTATCAGACACTGAG | MS2 | 0.1 | 216-300 | [18] |
|  | GTTGCTGGTCCCAATGTGATTTT |  |  |  |  |
| US9 | /5TET/GTGCATGTTAGCTGGTGCAA | MS2 | 0.1 | 140-238 | [18] |
|  | GTTGAGAACATGTCATTTGGGACT |  |  |  |  |
| US10 | /5HEX/GTTGGCTTGAAAAAGCCTTG | MS2 | 0.4 | 235-319 | [18] |
|  | GTTGCGACCTGCTCCATGTAAAA |  |  |  |  |
