# Supplementary material for: Population structure of Plains Sucker Pantosteus jordani (Evermann 1893) in Canada
Source: PLoS One. 2026 Aug 26;21(8):e0355547. doi: 10.1371/journal.pone.0355547 (PMC13514028; doi:10.1371/journal.pone.0355547)
Supplement: Supplementary file 3 [file pone.0355547.s003.docx]

**S3 Table.** GenBank accession numbers for the catostomid sequences used in the phylogenetic analyses.

|  |  | **Gene** | | | | | | |
| --- | --- | --- | --- | --- | --- | --- | --- | --- |
| **Species** | **SampleID** | **Complete mtDNA** | **atp** | **cytb** | **ND1** | **ND2** | **ND4** | **ND5/6** |
| *P jordani* | 227363 |  | KJ441226 | KJ441277 | KJ441379 | KJ441328 | KJ441175 | KJ441124 |
| *P jordani* | 227335 |  | KJ441225 | KJ441276 | KJ441378 | KJ441327 | KJ441174 | KJ441123 |
| *P bondi* | 227306 |  | KJ441222 | KJ441273 | KJ441375 | KJ441324 | KJ441172 | KJ441120 |
| *P bondi* | Simi1 |  | KJ441223 | KJ441274 | KJ441376 | KJ441325 | KJ441171 | KJ441121 |
| *P lahontan* | 112303 |  | KJ441219 | KJ441270 | KJ441372 | KJ441421 | KJ441168 | KJ441117 |
| *P lahontan* | 112324 |  | KJ441220 | KJ441271 | KJ441373 | KJ441322 | KJ441169 | KJ441118 |
| *P nebuliferous* | CATO78 |  | KJ441190 | KJ441241 | KJ441343 | KJ441292 | KJ441139 | KJ441088 |
| *P plebeius* | Rio1 |  | KJ441186 | KJ441237 | KJ441339 | KJ441288 | KJ441135 | KJ441084 |
| *P santaanae* | PSBT1 |  | KJ441211 | KJ441262 | KJ441364 | KJ441313 | KJ441160 | KJ441109 |
| *P clarkii* | FRA1 |  | KJ441207 | KJ441258 | KJ441360 | KJ441309 | KJ441156 | KJ441105 |
| *P virescens* | 57477 |  | KJ441214 | KJ441265 | KJ441367 | KJ441316 | KJ441163 | KJ441112 |
| *P platyrhynchus* | 114410 |  | KJ441199 | KJ441250 | KJ441352 | KJ441301 | KJ441148 | KJ441097 |
| *P discobolus* | HAV2 |  | KJ441195 | KJ441246 | KJ441348 | KJ441297 | KJ441144 | KJ441093 |
| *C insignis* | ecci 1 |  | KJ441232 | KJ441283 | KJ441385 | KJ441334 | KJ441181 | KJ441130 |
| *C ardens* | STR4CA |  | KJ441229 | KJ441280 | KJ441382 | KJ441331 | KJ441178 | KJ441127 |
| *C macrocheilus* | 227245 |  | KJ441228 | KJ441279 | KJ441381 | KJ441330 | KJ441177 | KJ441126 |
| *C columbianus* | 227313 |  | KJ441230 | KJ441281 | KJ441383 | KJ441332 | KJ441179 | KJ441128 |
| *C tahoensis* | Walk5 |  | KJ441231 | KJ441282 | KJ441384 | KJ441333 | KJ441180 | KJ441129 |
| *C catostomus* | MINCCAT |  | KJ441233 | KJ441284 | KJ441386 | KJ441335 | KJ441182 | KJ441131 |
| *C commersonii* | | AB127394 |  | | | | | |
| *Cycleptus dongatus* | | AB126082 |  | | | | | |
