# Supplementary material for: Population structure of Plains Sucker Pantosteus jordani (Evermann 1893) in Canada
Source: PLoS One. 2026 Aug 26;21(8):e0355547. doi: 10.1371/journal.pone.0355547 (PMC13514028; doi:10.1371/journal.pone.0355547)
Supplement: Supplementary file 4 [file pone.0355547.s004.docx]

**S4 Table.** Per locus observed (H_obs_) and expected (H_exp_) heterozygosity values for 371 plains sucker samples genotyped at eight microsatellite loci. A * denotes a significant difference between H_obs_ and H_exp_ after correction for multiple tests (p_crit_=0.0096).

| **Locus:** | **CCAT35** | | **CCAT44** | | **Dlu409** | | | **Dlu4283** | | | **US3** | | | **US4** | | | **US9** | | | **US10** | | |  |
| --- | --- | --- | --- | --- | --- | --- | --- | --- | --- | --- | --- | --- | --- | --- | --- | --- | --- | --- | --- | --- | --- | --- | --- |
| **Pop** | **H_obs_** | **H_exp_** | **H_obs_** | **H_exp_** | | **H_obs_** | **H_exp_** | | **H_obs_** | **H_exp_** | | **H_obs_** | **H_exp_** | | **H_obs_** | **H_exp_** | | **H_obs_** | **H_exp_** | | **H_obs_** | **H_exp_** | |
| **NSR** | 0.50 | 0.46 | 0.75 | 0.79 | | 1.00 | 0.96 | | 0.75 | 0.86 | | 0.75 | 0.82 | | 0.75 | 0.86 | | 0.50 | 0.46 | | 1.00 | 0.96 | |
| **CBR** | 0.90 | 0.94 | 0.70 | 0.79 | | 0.90 | 0.94 | | 0.44^*^ | 0.86 | | 0.80 | 0.86 | | 1.00 | 0.89 | | 0.70 | 0.76 | | 0.90 | 0.93 | |
| **HWC** | 0.94 | 0.86 | 0.84 | 0.81 | | 1.00 | 0.94 | | 0.47^*^ | 0.89 | | 0.71^*^ | 0.83 | | 0.94 | 0.92 | | 0.65 | 0.72 | | 0.87 | 0.93 | |
| **CNC** | 0.67 | 0.78 | 0.89 | 0.87 | | 0.78 | 0.90 | | 0.56^*^ | 0.92 | | 0.89 | 0.82 | | 1.00 | 0.91 | | 0.89 | 0.73 | | 1.00 | 0.89 | |
| **BR** | 0.69 | 0.77 | 0.79 | 0.81 | | 1.00 | 0.94 | | 0.69 | 0.90 | | 0.76 | 0.86 | | 0.90 | 0.89 | | 0.76 | 0.73 | | 0.93 | 0.91 | |
| **SMR** | 1.00 | 0.85 | 0.67 | 0.67 | | 1.00 | 0.95 | | 1.00 | 0.97 | | 1.00 | 0.85 | | 0.83 | 0.90 | | 0.67 | 0.55 | | 1.00 | 0.93 | |
| **LC** | 0.80 | 0.79 | 0.70 | 0.86 | | 0.86 | 0.93 | | 0.50^*^ | 0.92 | | 0.87 | 0.84 | | 0.93 | 0.92 | | 0.73 | 0.69 | | 0.93 | 0.91 | |
| **MR** | 0.98 | 0.94 | 0.66^*^ | 0.74 | | 0.92 | 0.94 | | 0.78^*^ | 0.88 | | 0.83 | 0.87 | | 0.87 | 0.91 | | 0.66 | 0.68 | | 0.86 | 0.93 | |
| **MR1** | 0.96 | 0.94 | 0.67 | 0.74 | | 0.89 | 0.96 | | 0.76 | 0.92 | | 0.81 | 0.85 | | 0.89 | 0.92 | | 0.78 | 0.70 | | 0.81 | 0.93 | |
| **MR2** | 1.00 | 0.93 | 0.60 | 0.72 | | 0.95 | 0.94 | | 0.86 | 0.89 | | 0.86 | 0.88 | | 0.76 | 0.93 | | 0.52 | 0.56 | | 0.95 | 0.93 | |
| **MR3** | 0.96 | 0.93 | 0.68 | 0.77 | | 0.93 | 0.93 | | 0.78 | 0.89 | | 0.82 | 0.88 | | 0.79 | 0.92 | | 0.68 | 0.72 | | 0.86 | 0.91 | |
| **MR4** | 0.96 | 0.94 | 0.71 | 0.65 | | 1.00 | 0.92 | | 0.64 | 0.87 | | 0.76 | 0.88 | | 0.96 | 0.90 | | 0.64 | 0.66 | | 0.79 | 0.93 | |
| **MR5** | 1.00 | 0.93 | 0.66 | 0.77 | | 0.86 | 0.93 | | 0.86 | 0.85 | | 0.90 | 0.87 | | 0.93 | 0.91 | | 0.66 | 0.74 | | 0.90 | 0.95 | |
| **BTC** | 0.91 | 0.84 | 0.72 | 0.74 | | 0.97 | 0.96 | | 0.91 | 0.95 | | 0.91 | 0.84 | | 1.00 | 0.95 | | 0.85 | 0.90 | | 0.94 | 0.93 | |
| **BTC1** | 0.86 | 0.86 | 0.57 | 0.67 | | 1.00 | 0.97 | | 1.00 | 0.92 | | 1.00 | 0.91 | | 1.00 | 0.92 | | 0.86 | 0.92 | | 1.00 | 0.96 | |
| **BTC2** | 1.00 | 0.96 | 1.00 | 0.93 | | 0.75 | 0.96 | | 0.50 | 0.82 | | 1.00 | 0.89 | | 1.00 | 0.96 | | 0.75 | 0.93 | | 1.00 | 0.93 | |
| **BTC3** | 1.00 | 0.86 | 0.73 | 0.78 | | 1.00 | 0.95 | | 1.00 | 0.96 | | 0.87 | 0.74 | | 1.00 | 0.96 | | 0.87 | 0.88 | | 1.00 | 0.95 | |
| **BTC4** | 0.71 | 0.77 | 0.71 | 0.67 | | 1.00 | 0.96 | | 0.86 | 0.95 | | 0.86 | 0.82 | | 1.00 | 0.95 | | 0.86 | 0.81 | | 0.71 | 0.87 | |
| **NMC** | 0.40 | 0.71 | 1.00 | 0.76 | | 1.00 | 0.98 | | 0.60 | 0.91 | | 1.00 | 0.87 | | 0.80 | 0.96 | | 1.00 | 0.98 | | 1.00 | 0.98 | |
| **CC** | 0.82^*^ | 0.88 | 0.62 | 0.69 | | 0.85^*^ | 0.91 | | 0.46^*^ | 0.87 | | 0.72 | 0.77 | | 0.88 | 0.91 | | 0.65 | 0.69 | | 0.87 | 0.91 | |
| **CGC** | 1.00 | 0.96 | 0.60 | 0.82 | | 1.00 | 0.98 | | 0.60 | 0.87 | | 0.80 | 0.71 | | 1.00 | 0.98 | | 0.40 | 0.76 | | 0.80 | 0.89 | |
| **YMT** | 0.82 | 0.91 | 0.56^*^ | 0.91 | | 1.00 | 0.93 | | 0.55 | 0.93 | | 0.82 | 0.93 | | 1.00 | 0.94 | | 1.00 | 0.95 | | 1.00 | 0.94 | |
